# CULTURAL AND SOCIAL ACTIVITIES AND MENTAL HEALTH AMONG INDIGENOUS OLDER ADULTS IN THE UNITED STATES

**DOI:** 10.1093/geroni/igac059.1910

**Published:** 2022-12-20

**Authors:** Yeonjung Lee, Kathryn Braun, Yanyan Wu, Collette Adamsen, Shelly Davis

**Affiliations:** University of Hawaiʻi at Mānoa, Honolulu, Hawaii, United States; University of Hawaiʻi at Mānoa, Thompson School of Social Work & Public Health, Honolulu, Hawaii, United States; University of Hawai‘i at Mānoa, Honolulu, Hawaii, United States; University of North Dakota, Grand Forks, North Dakota, United States; University of North Dakota School of Medicine & Health Sciences, Grand Forks, North Dakota, United States

## Abstract

Although the number and percentage of indigenous older adults are growing, little is known about the risk and protective factors associated with mental health among this population. We estimated regression models for mental health using data from the 2017-2020 needs assessment of indigenous elders (aged 55+) administered by the University of North Dakota, which included 19,143 indigenous elders, including 17,184 American Indians, 1,521 Alaska Natives, and 438 Native Hawaiians. The Mental Health Index (MHI) was used, with five questions: during the past month, how much of the time (1) were you a happy person, (2) have you felt calm and peaceful, (3) have you been a very nervous person, (4) have you felt downhearted and blue, and (5) have you felt so down in the dumps that nothing could cheer you up? The answers for MHI-5 were standardized on a 0-100 scale, where higher scores indicate optimal mental health. Having ADL and/or IADL difficulties was associated with worse mental health. Frequent engagement in social activities was positively associated with mental health. Data also suggest that those engaged in cultural practice all the time compared to people who engaged in cultural practice less frequently had higher levels of MHI-5, controlling for other variables. These findings underscore the importance of cultural activities, as well as social activities and physical health, in the management of mental health.

